# Effect of Mechanical Vibration on the Durability of Proton Exchange Membrane Fuel Cells

**DOI:** 10.3390/nano13152191

**Published:** 2023-07-27

**Authors:** Sitong Chen, Xueke Wang, Tong Zhu

**Affiliations:** 1 School of Mechanical Engineering, Shenyang University of Technology, Shenyang 110870, China; chensitong0525@126.com; 2 Beijing Institute of Space Launch Technology, Beijing 100076, China; 1810135@stu.neu.edu.cn; 3 School of Mechanical Engineering and Automation, Northeastern University, Shenyang 110819, China

**Keywords:** proton exchange membrane fuel cell, mechanical vibration, durability, platinum migration, accelerated stress test

## Abstract

To study the durability of proton exchange membrane fuel cells (PEMFCs), the experiments were performed by using a 300 h accelerated stress test under vibration and non-vibration conditions. Before and after chronic operation, the polarization curve, impedance spectra and cyclic voltammogram were measured at regular intervals. The voltage under vibration shows a small decline at the current density of 400 mA cm^−2^ and decreases quickly along the time in high current density. Meanwhile, the pavement vibration dramatically impacts the contact resistance of the membrane electrode assembly to the bipolar plates and the clamping screws of the fuel cell easily loosen under vibration. The calculations from X-ray diffraction patterns indicate that the average diameters of Pt particles under vibration are smaller than those under no-vibration conditions. It increases from 3.17 nm in the pristine state to 3.43 nm and 4.62 nm, respectively. Moreover, much more platinum that dissolved from the catalyst layer and redeposited was detected inside the polymer membrane under vibration conditions.

## 1. Introduction

The proton exchange membrane fuel cell (PEMFC) have become a new power option which can supersede the engines powered by fossil fuels in passenger vehicles owing to their characteristics of zero emissions, high efficiency and power density [1,2]. However, there are still challenges to fulfilling the target of 5000 hours’ steady operation for passenger vehicles presented by the US Department of Energy (DOE) [3].

In order to ensure better forecast accuracy of the fuel cell’s lifetime and analyze its probable degradation mechanism, the accelerated stress tests (ASTs) have been implemented by many researchers in experimental studies with regard to the contamination in hydrogen and air [4,5], start-up at subzero temperature [6,7,8,9] and dynamic response under driving cycles [10]. For the on-board fuel cells in vehicles, the performance degradation consists of membrane degradation, Pt/C catalyst degradation and gas diffusion layer degradation [11,12,13,14,15,16,17,18,19]. More specifically, the typical membrane degradation includes cracks, punctures and pinholes, resulting from a harsh operating environment accompanied by improper temperature, relative humidity and mechanical conditions. In the long run, rupture, delamination, agglomeration and migration of Pt or carbon corrosion will all result in the Pt/C catalyst degradation. All of these negative effects, which arise from either the changes in the microstructure of carbon-supported platinum or the decline of electrons and ions involved in the electrochemical reaction, lead to an obvious loss of catalyst activity. Additionally, the gas diffusion layer degradation due to the poly-tetrafluoroethylene (PTFE) decomposition, the movement of micropores and the loss of microporous layer (MPL) hydrophobicity caused by the carbon corrosion-based degradation process often cause the variation of water content.

Even though the degradation phenomenon of PEMFC has been investigated, research on the performance degradation of vehicle fuel cell stacks subjected to vibration situations is in progress. Among the few open literatures, Imen et al. [20] evaluated the effect of mechanical loads and vibrations on an open-cathode PEMFC in operating state as well as a non-operating PEMFC; the experimental results reveal that these external factors can change the performance and reliability of the fuel cell by causing physical damage to the fuel cell components. Rajalakshmi et al. [21] performed vibration test analysis on a 500 W PEM fuel cell stack developed by simulating some application situations in the stack and evaluated the robustness of the stack; the fuel cell performance was in good agreement before and after the vibration and shock tests, showing the mechanical integrity of the system. Hou et al. [22] studied the performance of a fuel cell stack through long-term strengthened road vibration tests, the individual cell voltage uniformity became distinctly worse. With the increase of vibration duration, the ohmic resistance obtained from AC impedance diagnosis ascended approximately linearly and presented a growth of 5.36% ultimately. Wu et al. [23] numerically analyzed the mechanical response of a large fuel cell stack clamped by steel belts to a violent impact. The results indicate that the stack may give rise to interface slippage between cells when subjected to a large impact in the direction parallel with the cells, showing a downward bowing phenomenon. Wang et al. [24] experimentally investigated the effect of mechanical vibration on the dynamic response of PEMFC. Diloyan et al. [25] discussed the effect of mechanical vibration on platinum particle agglomeration and growth in the catalyst layer of PEMFC; it was observed that the average diameter of Pt particles under vibration was 10% smaller than the ones that were under no-vibration conditions.

In the current research, the influence of vibration on the durability of PEMFCs was studied, presenting the results of the performance variation in PEMFCs. A 300 h vibration test was accomplished to assess the effect of vibration as for the endurance of MEA and the results were characterized clearly by electrochemical and physical methods. The polarization curve, impedance spectra and cyclic voltammogram were measured at regular intervals. Meanwhile, the changes in the microstructure of MEAs were observed and measured by X-ray Diffraction (XRD), Scanning Electron Microscopy (SEM) and Transmission Electron Microscopy (TEM) before and after the experiments.

## 2. Experimental Section

### 2.1. Experimental Setup

**Figure 1:**
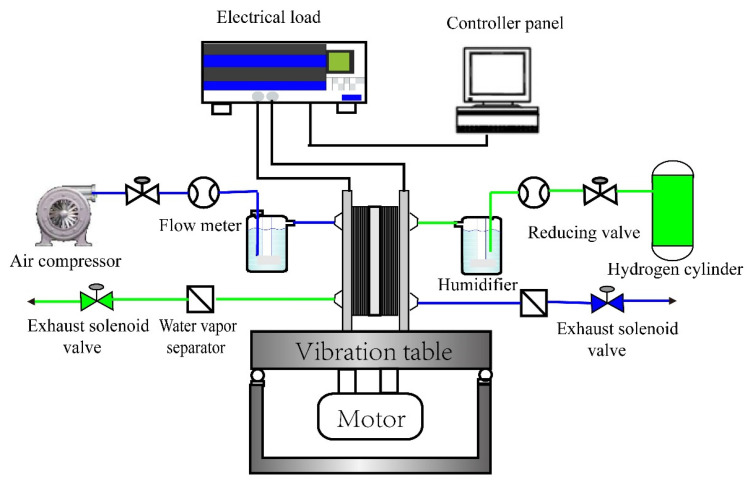
The schematic illustration of the test rig.

In Figure 1, the experimental bench is composed of the vibration generator, the electronic load, the data acquisition system, the bubble humidifier and the gas supply system. The vibration tests of PEMFC were performed on the horizontal and vertical vibration generator controlled by computer software, which could produce excitations of multiple waveforms within the frequency scope of 1 Hz to 600 Hz with the maximum displacement of 5 mm. Its acceleration amplitude is up to 20 g and the maximum load is 100 kg.

As to PEMFC, the parallel serpentine flow channel of 25 cm^2^ was used for the experiments. For MEA, a commercially available Nafion^®^ 211 membrane and SGL-25BC carbon papers, of which the porosity was 80% and the air permeability was 1.0 cm^3^/(cm^2^·s), were chosen. The Pt catalyst loading on the cathode side and anode side were, respectively, 0.48 mg cm^−2^ and 0.28 mg cm^−2^.

### 2.2. Test Procedure

Based on the typical vibration feature of running vehicles and the actual road conditions, the accelerated stress test was designed to research the durability of PEMFCs under both the cases of vibration and non-vibration. The vibrational frequency of vehicles on the road is close to 17–40 Hz and the maximum vibration acceleration of the vehicles in the horizontal and vertical directions are usually less than 2.06 g and 4.665 g, respectively [26]. Therefore, the vibration experiments were conducted at the frequency of 20 Hz and the horizontal acceleration and vertical acceleration of 2.0 g. During an experimental cycle, the vibration generator first provided the horizontal excitation for 20 min with a rest for 5 min, and then produced the vertical excitation for 20 min. For the purpose of analyzing the influence of mechanical vibration on the durability of PEMFC, the PEMFC was operated under vibrational and static conditions, along with the operating parameters summarized in Table 1.

Throughout the vibration tests, the performance of PEMFC was evaluated several times at regular intervals by measuring the polarization curves, electrochemical impedance spectroscopy (EIS) and cyclic voltammogram (CV). For the EIS measurements, the test frequency was chosen in the range of 10 kHz to 10 mHz while the amplitude was kept at 5 mV. Moreover, the scanning rate of CV was recorded at 50 mV/s.

### 2.3. Characterization of MEA

The micromorphology characteristics and the changes in the microstructure of MEAs were observed and measured by X-ray Diffraction (XRD), Scanning Electron Microscopy (SEM) and Transmission Electron Microscopy (TEM) before and after the experiments. In order to compare the Pt grain diameter of the cathode side with TEM, we first scraped some Pt/C catalyst off the MEA and dissolved it in the ethanol–water mixture, and then split the Pt particles from the carbon support with ultrasonic shaking for 2 h. Afterwards, 1 mL mixture was extracted and dripped onto the copper mesh for observing.

## 3. Results and Discussion

**Figure 2:**
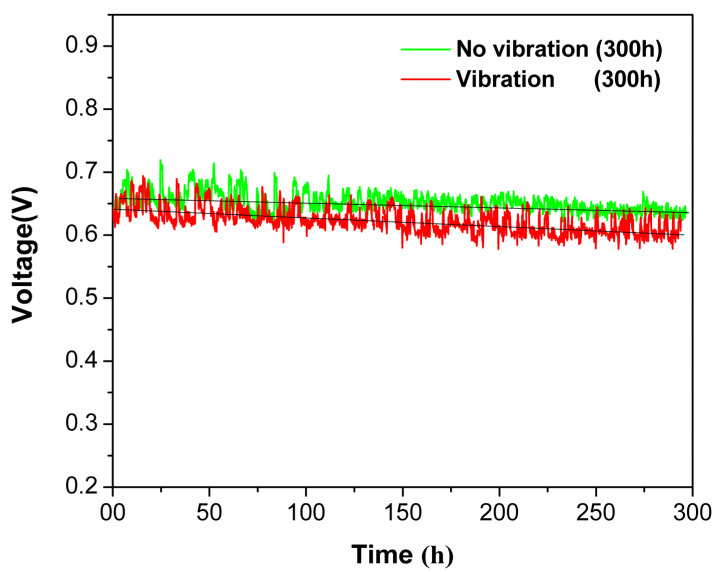
The effect of vibration on voltage degradation of the PEMFC operated at 400 mA cm^−2^.

The degradation of the PEMFC running continuously for 300 h under vibrational and static conditions is exhibited in Figure 2. Under vibration conditions, the cell voltage drops with a faster rate during the 300 h operation, the average performance of the PEMFC decreases from 0.643 V to 0.612 V and the degradation rate reaches 103 μVh^−1^. While running at no-vibration conditions, a voltage difference from 0.658 V to 0.642 V is indicated and the voltage decay rate is approximately 53 μVh^−1^. The U.S. Department of Energy sets the stack voltage at the end of lifetime as 90% of the initial voltage at the rated power output [3], and the degradation rate is assumed to be constant based on the results of relevant studies [22,27]. It can be inferred that the durability of the MEA under static condition can reach 1500 h, which only reaches 700 h under vibration conditions. The voltage drop is one of the manifestations of the vehicular PEMFCs’ performance degradation caused by chronic pavement vibration. In addition, the voltage fluctuates greatly under vibration conditions, which is an interruption phenomenon caused by instability in the PEM fuel cell operation under vibration.

**Figure 3:**
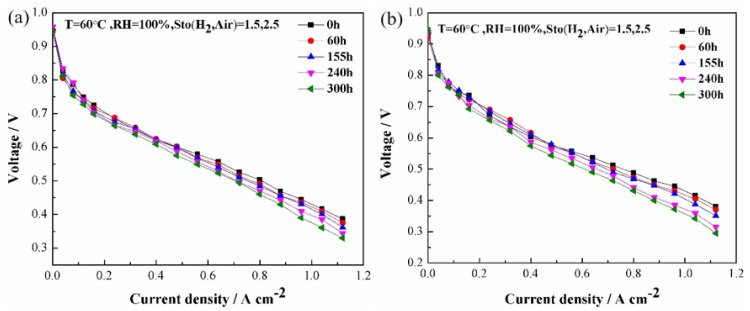
The polarization curves of a 25 cm^2^ single cell under (**a**) no-vibration and (**b**) vibration conditions.

Figure 3a,b present the polarization curves of the PEMFC operating at different duration under vibration and no-vibration conditions, respectively. The kinetics region remains almost unaltered, and the ohmic region seems to slightly degrade compared to the non-vibration experiments. With the growth of current density, the attenuation ratio of voltage that represents the slope of the voltage value before and after the decay under the increase of the unit current density becomes larger under both cases. Particularly when running for 240 h at no-vibration conditions, the performance of the cell remains stable with the current density less than 400 mA cm^−2^, but in the mass-transfer region density the voltage gradually falls off; the mass-transfer region is the most affected section of the polarization curves, indicative of a degradation of the electrode structure. Compared with above results, the voltage under vibration has shown a small decline at the current density of 400 mA cm^−2^ and decreases faster along the time in the high-current-density region.

**Figure 4:**
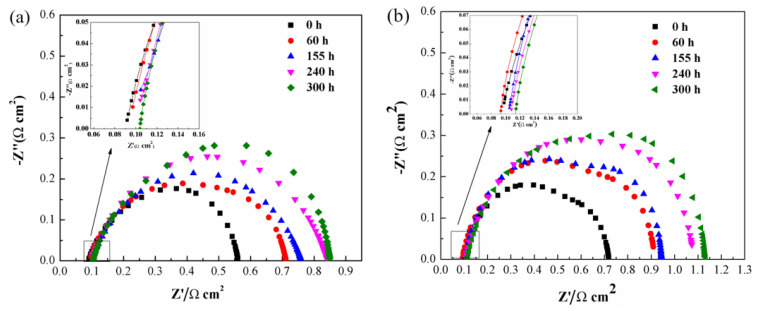
The Nyquist plots of a 25 cm^2^ single cell measured at 400 mA cm^−2^ under (**a**) no-vibration and (**b**) vibration conditions.

For all the single PEMFC, the high frequency intercept denotes the sum of interfacial contact and material bulk resistance (Rohm). The medium frequency arc reflects the combination of a charge transfer resistance (Rct) and a double layer capacitance (C) within the catalyst layer. The low frequency arc represents the mass transport process [28]. As is revealed in Figure 4a,b, the impedance loop of the PEMFC increases with the extension of discharge time, the ohmic resistance of PEMFC at no-vibration conditions increases from 0.09 Ω cm^−1^ to 0.102 Ω cm^−1^, while the ohmic resistance under vibration enhances from 0.09 Ω cm^−1^ to 0.114 Ω cm^−1^. This indicates that the pavement vibration dramatically impact the contact resistance of the membrane electrode assembly to the bipolar plates and the clamping screws of the fuel cell easily loosen under vibration conditions.

**Figure 5:**
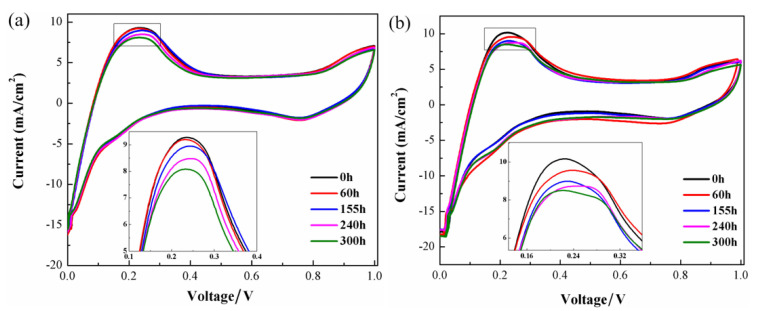
The cyclic voltammograms of a 25 cm^2^ single cell operated under (**a**) no-vibration and (**b**) vibration conditions.

The cyclic voltammograms measured after running at different test duration under vibrational and static conditions are presented in Figure 5. Obviously, the oxidation desorption peak of hydrogen is observed at about 0.2 V and the peak area decreases with the increase of operating time under both cases. The electrochemical active surface area (ECSA) can be calculated with the following equation [29]:

(1) $$\mathrm{ECSA}=\frac{\left[\mathrm{Charge}\;\mathrm{area}/V\right]\times 1000}{0.21\;\times \;\mathrm{Catalyst}\;\mathrm{loading}\times 10000\;\;}\;$$

**Figure 6:**
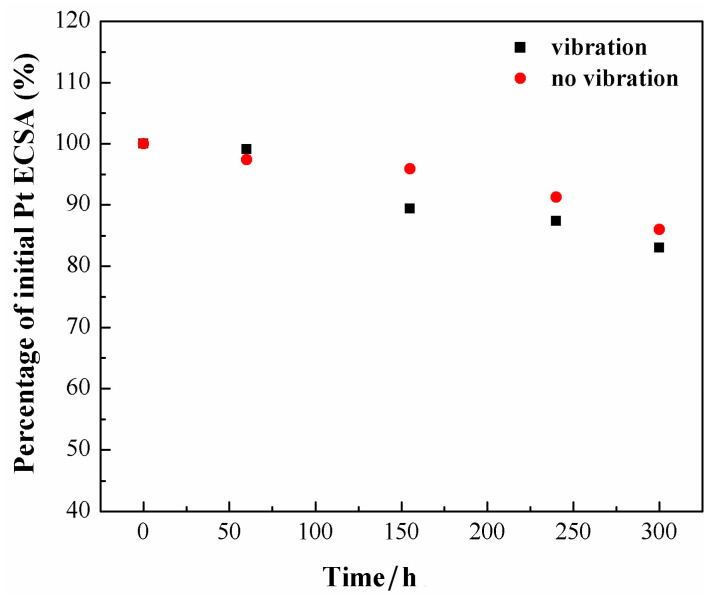
The variation of the effective active area of catalyst layer with running time.

As is shown in Table 2, ECSA reduces with the increase of operating time, which changes between 60.1 and 49.6 m^2^ g^−1^, respectively, under vibration conditions and falls from 57.5 to 50 m^2^ g^−1^ under static conditions. In Figure 6, the ratio between the effective active area and the initial active area after different running times is plotted. It can be observed that the effective active area has decreased greatly in the vibrational environment since the operation time of 150 h and the drop degree of the ratio under vibration is always greater than that under no-vibration conditions. The decline of ECSA to the catalyst may be on account of the agglomeration and dissolution of Pt nanoparticles, as well as the detachment of Pt nanoparticles from the carbon support.

**Figure 7:**
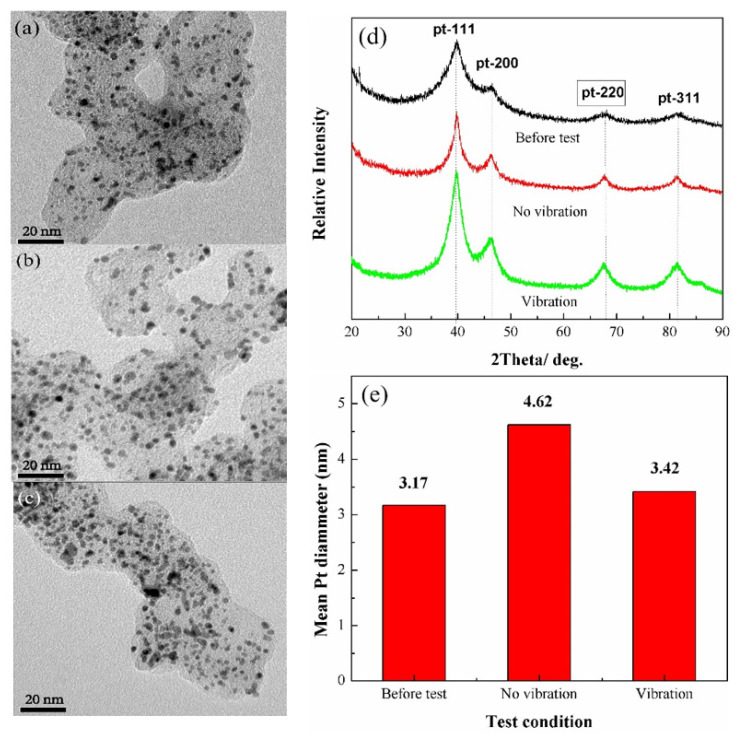
TEM micrographs of the carbon-supported Pt catalyst layer on the cathode side (**a**) before test, under (**b**) no-vibration and (**c**) vibration conditions after 300 h accelerated test. (**d**) X-ray diffraction patterns of Pt/C on the cathode side for the three cases; (**e**) the average particle size of Pt/C calculated from XRD analysis.

The microscopic state of Pt particles on the cathode side of MEA was analyzed with TEM in terms of the state before the test, and under no-vibration and vibration conditions after 300 h accelerated test, as shown in Figure 7a–c. From the obtained TEM images, we can see that after the static and vibrational tests, the crystal particles of Pt become larger and agglomerated, and then the overall uniformity of the particles are worse than that before the test. In order to confirm and quantify the particle size distribution, X-ray scattering measurements were executed to calculate the average particle size. As can be seen from X-ray diffraction patterns, the characteristic diffraction peaks of Pt at 2θ = 40°, 46°, 68° and 81°, respectively, belong to the (111), (200), (220) and (311) crystal planes. Before the experiments, the diffraction peaks of the catalyst showed a wide broadened full-width at half of maximum, indicating that the grain diameter of Pt is smaller compared with under vibration and no-vibration conditions. Due to the diffraction intensity of Pt peaks, (111), (200) and (311) are interfered by the carbon-10 and carbon-11 characteristic peaks, and the Pt (220) crystal plane was used to calculate the average grain size of the catalyst with Sherrer formula [30]:

(2) $$d=\frac{0.9\lambda_{\mathrm{ka}}}{B_{2\theta }\cos\theta_{B}}$$

where d represents the average grain diameter of the catalyst, λ_ka_ is 1.54056 Å, θ_B_ represents the diffraction angle of the Pt-220 crystal plane and B_2θ_ represents the full width at half of maximum.

The average grain diameter of the catalyst is summarized in Figure 7e. The grain diameter before the test was 3.17 nm; after 300 h of operation under vibration and no-vibration conditions, the grain diameter of catalyst enhanced to 3.43 nm and 4.62 nm, respectively. It can be observed that the Pt particles on the cathode side have different extents of agglomeration after chronic operation under both cases in comparison to the state before the test. The catalyst agglomeration will bring about the drain in the ECSA, leading to degradation in the performance of PEMFC [15,31], but the grain diameter of a catalyst under vibration is smaller than that in a static environment, and so the Pt particles’ agglomeration is not the only reason for the vehicular PEMFCs’ performance degradation after chronic pavement vibration.

**Figure 8:**
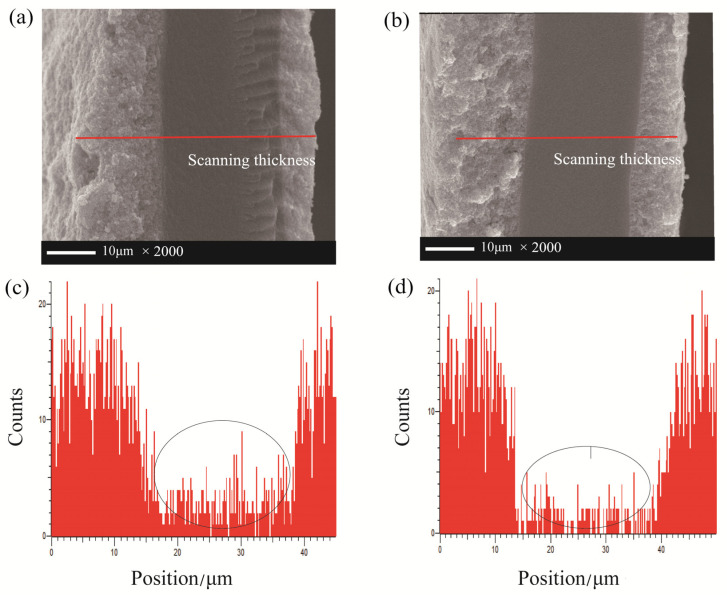
EDX element analysis of Pt after 300 h accelerated tests: (**a**) the cross-section of MEA and (**c**) EDX element analysis under vibration; (**b**) the cross-section of MEA and (**d**) EDX element analysis under no-vibration conditions.

To further explain the attenuation mechanism of MEA in a vibration environment, EDX element analysis is performed on the cross-section of MEA at the end of 300 h accelerated tests in vibration and no-vibration environments. From Figure 8a,b, the cross-section of MEA shows that the interfaces of the catalyst layer to the membrane are very flat and without delamination under vibration and static conditions. Furthermore, it can be seen in Figure 8c,d that after a long time of operation, the polymer membrane which contains no platinum element shows the existence of platinum under both cases. This illustrates that the dissolved Pt ions from the cathode side transfer into the polymer membrane along the hydrophilic channels and redeposit in it, which also give rise to the drain of ECSA [32,33,34]. Meanwhile, much more platinum is detected inside the proton exchange membrane under vibration conditions than static conditions. The Pt catalyst loading of both cases before the tests are equivalent, so that more Pt particles drain from the catalyst layer, resulting in greater loss of the electrochemical active area and the performance of PEMFC.

## 4. Conclusions

In the present study, we demonstrate that the voltage degradation rate is 103 μV h^−1^ under vibration conditions during a 300 h accelerated test, which is higher than the 53 μV h^−1^ degradation rate under no-vibration conditions. The high frequency resistance under vibration conditions increases faster in the test time. This is by reason of the clamping screws of the PEMFC easily loosen under vibration conditions. In addition, according to TEM images and XRD patterns, the Pt particles on the cathode side show different extents of agglomeration after chronic operation under both cases, which lead to degrade in the performance of PEMFC. To explain why the performance and the electrochemical active area of the PEMFC under vibration operation remain a faster decrease than those of the PEMFC under static environment, EDX element analysis was performed. Much more platinum that dissolved from the catalyst layer and redeposited was detected inside the polymer membrane under vibration conditions, resulting in greater loss of the electrochemical active area and the performance of PEMFC.

## Figures and Tables

**Table 1:** The test conditions of the fuel cell.

Test Conditions	
Fuel/Oxidant	Hydrogen/Air
Constant current density	400 mA/cm^2^
PEMFC temperature	65 °C
Anode/Cathode humidity	100%/100%
Anode/Cathode stoic	1.5/2.5
Test length	300 h

**Table 2:** The ECSA of MEA with operating time under vibration and no-vibration conditions.

Operating Time	Under Vibration		Under No Vibration	
	Peak Area (mC cm^−2^)	ECSA (m^2^ g^−1^)	Peak Area (mC cm^−2^)	ECSA (m^2^ g^−1^)
0 h	60.6	60.1	58	57.5
60 h	60.2	59.7	56.4	55.9
155 h	54.4	53.9	55.6	55.1
240 h	52	51.5	52.6	52.1
300 h	50	49.6	50.4	50

## Data Availability

The data presented in this study are available in this article.
